# The role of Rab27 in tick extracellular vesicle biogenesis and pathogen infection

**DOI:** 10.1186/s13071-024-06150-7

**Published:** 2024-02-09

**Authors:** L. Rainer Butler, Nisha Singh, Liron Marnin, Luisa M. Valencia, Anya J. O’Neal, Francy E. Cabrera Paz, Dana K. Shaw, Adela S. Oliva Chavez, Joao H. F. Pedra

**Affiliations:** 1 The University of Maryland Baltimore, Baltimore, MD USA; 2 Washington State University, Pullman, WA USA; 3 Texas A&M University, College Station, TX USA; 4 Present Address: Harvard Medical School, Boston, MA USA; 5 Present Address: Memorial Sloan Kettering Cancer Center, New York, NY USA

**Keywords:** Ticks, Extracellular vesicles, Tick-borne diseases, Rab27

## Abstract

**Background:**

The blacklegged tick, *Ixodes scapularis*, transmits most vector-borne diseases in the US. It vectors seven pathogens of public health relevance, including the emerging human pathogen *Anaplasma phagocytophilum*. Nevertheless, it remains critically understudied compared to other arthropod vectors. *Ixodes scapularis* releases a variety of molecules that assist in the modulation of host responses. Recently, it was found that extracellular vesicles (EVs) carry several of these molecules and may impact microbial transmission to the mammalian host. EV biogenesis has been studied in mammalian systems and is relatively well understood, but the molecular players important for the formation and secretion of EVs in arthropods of public health relevance remain elusive. RabGTPases are among the major molecular players in mammalian EV biogenesis. They influence membrane identity and vesicle budding, uncoating, and motility.

**Methods:**

Using BLAST, an in silico pathway for EV biogenesis in ticks was re-constructed. We identified Rab27 for further study. EVs were collected from ISE6 tick cells after knocking down *rab27* to examine its role in tick EV biogenesis. *Ixodes scapularis* nymphs were injected with small interfering RNAs to knock down *rab27* and then fed on naïve and *A. phagocytophilum*-infected mice to explore the importance of *rab27* in tick feeding and bacterial acquisition.

**Results:**

Our BLAST analysis identified several of the proteins involved in EV biogenesis in ticks, including Rab27. We show that silencing *rab27* in *I. scapularis* impacts tick fitness. Additionally, ticks acquire less *A. phagocytophilum* after *rab27* silencing. Experiments in the tick ISE6 cell line show that silencing of *rab27* causes a distinct range profile of tick EVs, indicating that Rab27 is needed to regulate EV biogenesis.

**Conclusions:**

Rab27 is needed for successful tick feeding and may be important for acquiring *A. phagocytophilum* during a blood meal. Additionally, silencing *rab27* in tick cells results in a shift of extracellular vesicle size. Overall, we have observed that Rab27 plays a key role in tick EV biogenesis and the tripartite interactions among the vector, the mammalian host, and a microbe it encounters.

**Graphical Abstract:**

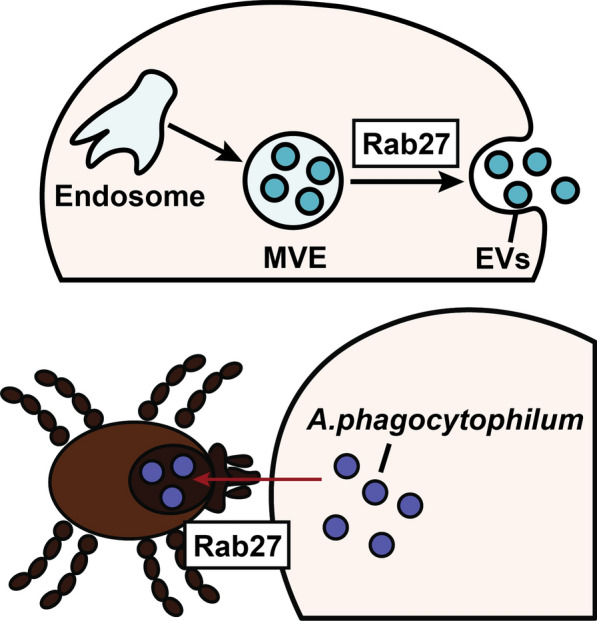

**Supplementary Information:**

The online version contains supplementary material available at 10.1186/s13071-024-06150-7.

## Background

The most medically relevant arthropod vector in the US is *Ixodes scapularis*, the blacklegged or deer tick [1]. *Ixodes scapularis* is responsible for spreading seven known human pathogens, encompassing bacteria, viruses, and parasites. In 2019, the Centers for Disease Control and Prevention (CDC) reported over 50,000 cases of tick-borne illness including 34,000 cases of Lyme disease and over 5000 cases of human granulocytic anaplasmosis [2]. Current estimates have calculated that Lyme disease alone costs the US economy around $345 to $968 million per year [3]. Furthermore, the burden of vector-borne diseases on the healthcare system is expected to increase in the coming years [4–7].

Ticks are hematophagous or blood-feeding arthropods. When a tick takes a blood meal, it introduces its saliva into the wound along with any microbes it is carrying. Tick saliva has long been studied and is known to contain a variety of anesthetics, anti-coagulants, and anti-inflammatory compounds [8–11]. However, the mechanism of delivery of these compounds was unknown until recently. It was found that tick cells secrete extracellular vesicles (EVs) in their saliva [12]. Tick EVs have been shown in microbial transmission and to enable arthropod fitness [12–14]. Despite their role in arthropod fitness and disease, the molecular mechanisms and proteins involved in tick extracellular vesicle biogenesis remain poorly defined.

The bulk of EV biogenesis research has been completed in mammalian systems. In mammals, a variety of proteins participate in forming EVs including endosomal sorting complex required for transport (ESCRT) proteins, tetraspanins, soluble N-ethylmaleimide-sensitive factor attachment receptor (SNAREs), and Rab-GTPases [15]. These proteins aid in the transition from early to late endosome and the invagination of the endosome, which creates intraluminal vesicles (ILVs) and the multivesicular endosome (MVE) [16–18]. The MVE is then trafficked to and fuses with the plasma membrane, releasing EVs into the extracellular space. Once EVs are released, they are known to function in cell-to-cell communication, by transporting a variety of molecules. Additional evidence shows that components of the EV biogenesis pathway can be manipulated by intracellular bacteria and are important for transmission of arthropod-borne pathogens in other systems [12, 13, 19–21]. The function of EVs has not been thoroughly explored in arthropod biology. Although it is known that ticks and tick cells secrete EVs, how EV biogenesis occurs in ticks remains elusive. Here, we indicate that silencing *rab27* in *I. scapularis* impacts arthropod fitness and microbial infection.

## Methods

### Mice and ticks

C57BL/6 J mice were obtained from the University of Maryland Veterinary Resources. All mice used were between 6 and 8 weeks of age. *Ixodes scapularis* nymphs were supplied by the Oklahoma State University and the University of Minnesota breeding colonies. Ticks were housed upon arrival at 23 °C with 85% relative humidity and a 14/10-h light/dark photoperiod cycle. All mouse experiments were performed according to the protocols approved by the Institutional Biosafety (IBC-00002247) and Animal Care and Use Committee (IACUC-01121014) at the University of Maryland School of Medicine and complied with National Institutes of Health (NIH) guidelines (Office of Laboratory Animal Welfare [OLAW] assurance number A3200-01).

### Tick cell culture

The *I. scapularis* ISE6 cell line was cultured in L15C300 medium supplemented with 10% heat-inactivated fetal bovine serum (FBS, 24 MilliporeSigma), 10% tryptose phosphate broth (BD), and 0.1% bovine lipoprotein concentrate (MP Biomedicals) at 34 °C [22]. Tick cells were grown to confluence and sub-cultured in capped T25 flasks (Greiner bio-one). All cell cultures were verified by PCR to be *Mycoplasma*-free (Southern Biotech).

### *Anaplasma phagocytophilum* culture

*Anaplasma phagocytophilum* strain HZ was cultured in vented T25 flasks (CytoOne) containing HL-60 cells. HL-60 cells were cultured in 20 ml RPMI medium, supplemented with 10% fetal bovine serum and 1 × Glutamax. *Anaplasma phagocytophilum*-infected cells (500 μl) were added to 5 ml of uninfected cells at 1 to 5 × 10^6^ cells/ml diluted in 24.5 ml of media. Infection percentage was monitored by the Richard-Allan Scientific™ three-step staining (Thermo Fisher Scientific). Infected cells were spun onto microscope slides with a Cytospin (Thermo Scientific). Cells were visualized by light microscopy with an Axioskop microscope (Zeiss). Bacteria were either used for experiments or sub-cultured once cultures had reached > 90% infection. Bacterial numbers were estimated using the number of infected HL-60 cells × 5 morulae/cell × 19 bacteria/cell.

### Bioinformatics

Tick homologs of protein sequences involved in EV biogenesis in humans, mice, and *Drosophila melanogaster* were identified and compared using the NIH Basic Local Alignment Search Tool for proteins (BLASTp). Proteins with Expect (E) values < 1 × 10^–5^ and > 80% query coverage were selected. The homologs identified were used to construct a putative EV biogenesis pathway for ticks.

### RNA interference

Small interfering RNAs (siRNAs) and scramble RNAs (scRNAs) were designed based on the sequence of tick *rab27* that was identified during the construction of the in silico EV biogenesis pathway in ticks. siRNAs were designed using BLOCK-iT RNAi designer (https://rnaidesigner.thermofisher.com/rnaiexpress/), and scRNA was designed using InvivoGen siRNA Wizard Software (https://www.invivogen.com/sirnawizard/scrambled.php). Both siRNAs and scRNAs were blasted against the *I. scapularis* genome to confirm specificity and avoid off target effects. All siRNA primers can be found in Additional file 1: Table S1.

For in vitro experiments, si-*rab27* and sc-*rab27* were synthesized by MilliporeSigma with dTdT overhangs. ISE6 cells were plated at 5 × 10^5^ cells per well (24 well plate) or 1 × 10^6^ cells per well (6 well plate). siRNAs (1 μg per ml) were nucleofected into ISE6 cells using the 4D-Nucleofector System (Lonza Bioscience). Tick cells were centrifuged at 100 × g for 10 min to pellet the cells. The pellet was washed with 10 ml dPBS, resuspended in SF buffer (Lonza Bioscience), and si-*rab27* or sc-*rab27* was added to the suspension. The nucleofection mix was added to a multiwell cuvette, inserted into the nucleofector, and pulsed using pulse condition EN150. The cells were then rested in the cuvette for 10 min post-nucleofection before being added to pre-warmed L15C300 complete media and seeded for experiments.

For in vivo experiments, si-*rab27* and sc-*rab27* were synthesized using the Silencer siRNA construction kit (Thermo Fisher Scientific) using the primers in Additional file 1: Table S1. *Ixodes scapularis* nymphs were microinjected with 20–40 ng of si-*rab27* or sc-*rab27* RNA in a 50 nl volume. Nymphs were rehoused and returned to the incubator and rested for 24 h before placement on mice.

### EV-depleted medium

L15C300 medium was supplemented with 5% fetal bovine serum (MilliporeSigma), 5% tryptose phosphate broth (BD), and 0.1% lipoprotein concentrate (MP Biomedicals). Medium was cleared from EVs by ultracentrifugation at 100,000 × g for 18 h at 4 °C in a LE-80 ultracentrifuge (Beckman Coulter) with a 60Ti rotor [ 13]. The absence of EVs was confirmed by determining the particle size distribution with a NanoSight NS300 (Malvern Panalytical) for nanoparticle tracking analysis (NTA). If EVs were present, the medium was subjected to a second ultracentrifugation at 100,000 ×g for 18 h at 4 °C. EV-free medium was sterilized by passing the content through a 0.22-μm Millipore Express^®^ PLUS (MilliporeSigma).

### EV collection

ISE6 cells were nucleofected with si-*rab27* or sc-*rab27* as described above. After 72 h, the L15C300 medium was replaced with EV-depleted L15C300 medium. EV collection was carried out as described previously [13]. Briefly, the EV-depleted medium was collected from cell cultures and cleared of any live cells by centrifugation at 300 × g for 10 min at 4 °C. Live cell pellets were resuspended in 1 ml Trizol and saved for RNA extraction and qPCR. Dead cells were removed by a second centrifugation at 2000 × g for 10 min at 4 °C. The supernatant was collected, and apoptotic bodies were removed by a third centrifugation at 10,000 × g for 30 min at 10 °C. To reduce the number of EVs > 200 nm in size, the supernatant was filtered through a 0.22-μm Millipore syringe filter (Millipore-Sigma). EVs were pelleted by ultracentrifugation (100,000 × g) for 18 h at 4 °C. Supernatant was discarded, and EVs were resuspended in 1:500 in 1 × PBS for NTA analysis.

### EV quantification

EV concentration and sizes were determined using a NanoSight NS300 (Malvern Panalytical) with NTA software version 3.0. The mean of the size generated in the NTA reports was used to calculate the average size of the EVs in each sample. Data were analyzed using GraphPad Version 10.0.3 from Prism.

### Quantitative reverse transcriptase polymerase chain reaction (qRT-PCR)

The PureLink RNA Mini kit (Invitrogen) was used to extract RNA from cells or ticks in Trizol. cDNA was synthesized with the Verso cDNA Synthesis Kit (Thermo Fisher Scientific). qRT-PCR was performed with the CFX96 Touch Real Time PCR Detection System (Biorad). For bacterial acquisition experiments, *A. phagocytophilum 16S rRNA* gene expression was measured by absolute quantification and normalized to *I. scapularis* actin. Copy numbers for *A. phagocytophilum* and *I. scapularis* were calculated from a standard curve. Tick *rab27* gene expression was measured by relative quantification and normalized to *I. scapularis* actin. The fold changes in gene expression were calculated using the 2 − ΔΔC T method. Non-template controls were included to confirm the absence of primer-dimers and contamination. Gene expression was measured using iTaq Universal SYBR Green Supermix (Bio-Rad Laboratories). Amplifications were done using the following conditions: an initial denaturation cycle at 95 °C for 5 min, followed by 35 cycles of denaturation for 15 s at 95 °C, amplification at three different annealing temperatures (*rab27*: 58 °C; *Actin*: 57 °C; *A. phagocytophilum 16S*: 54 °C) for 30 s, and extension at 72 °C for 30 s. Primers were used at a final concentration of 400 nM each, and 2 μl of cDNA was used as template. The specificity of the products was determined by single peaks in the melting curves, and each control and sample was run in duplicate. Primers were designed using the PrimerQuest Tool (IDT). All primers used in this study can be found in Additional file 1: Table S1.

### Tick fitness experiments

*Ixodes scapularis* nymphs were microinjected with 20–40 ng of si-*rab27* or sc-*rab27* in a 50 nl volume. Nymphs were rested for 24 h. After 24 h, the silenced or scrambled nymphs were placed on separate C57BL/6 J mice for 20 min and allowed to feed for 3 days. Ticks were removed from mice with forceps (partially engorged). Fully engorged ticks were not considered in these experiments. The weight of the ticks was measured using a Pioneer™ analytical balance (OHAUS), and engorgement was evaluated. Ticks labeled as having a weight of zero were below the scale’s limit of detection of 1 mg. Ticks were stored at −80 °C until RNA extraction.

### *Anaplasma phagocytophilum* acquisition experiments

One week prior to nymph placement, 6–8-week-old male C57BL/6 J mice were intraperitoneally (i.p.) injected with HL-60 cells containing *A. phagocytophilum* (1 × 10^7^ bacteria/injection) resuspended in 100 μl 1× PBS. At 6 days post infection (d.p.i.), *I. scapularis* nymphs were microinjected with 20–40 ng of si-*rab27* or sc-*rab27*. Nymphs were rested for 24 h. At 7 d.p.i., the silenced or scrambled nymphs were placed on C57BL/6 J for 20 min and allowed to feed for 3 days. Ticks were removed from mice with forceps, and fallen ticks were recovered from a water trap. Tick attachment was calculated based on contingency using Fisher’s exact test. The weight of the ticks was measured using a Pioneer^™^ analytical balance (OHAUS), and tick engorgement was evaluated. Ticks were stored at −80 °C until RNA extraction.

### Statistical analysis

Statistical significance of *rab27* silencing, vesicle concentration, and weight were assessed with an unpaired *t*-test with Welch’s correction. Tick attachment was calculated based on contingency using a Fisher’s exact test. We used GraphPad PRISM^®^ (GraphPad Software version 10.0.3) for all statistical analyses. Outliers were detected by a Graphpad Quickcalcs program (https://www.graphpad.com/quickcalcs/Grubbs1.cfm).

## Results

### Building an in silico extracellular biogenesis pathway in *I. scapularis*

**Fig. 1:**
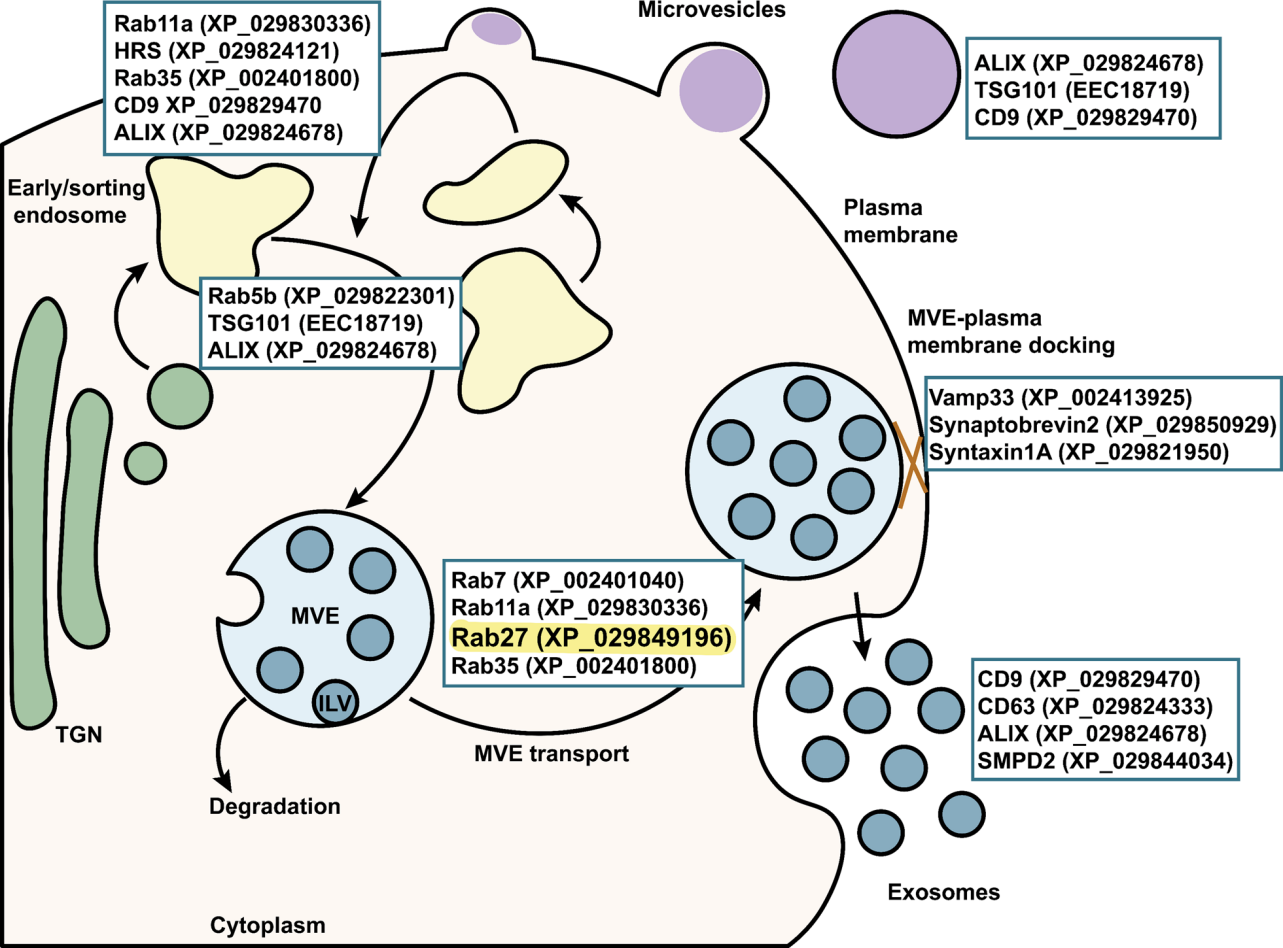
In silico analysis of EV biogenesis in the *Ixodes scapularis* genome. Sequences from proteins involved in mammalian EV biogenesis were input into the NCBI Basic Local Alignment Search Tool for proteins (BLASTp) against the *I. scapularis* genome and arranged based on mammalian literature to construct an in silico pathway in ticks. Accession numbers are listed in parentheses. Rab27 is highlighted

Using BLAST, we found several groups of proteins associated with human EV biogenesis including Rab-GTPases, SNARE, ESCRT-dependent and -independent pathway, and tetraspanin proteins (Fig. 1, Additional file 1: Table S2). Prior research in *I. scapularis* involved *Vamp33*, a SNARE protein important for exosome release [13], which is located at the terminus of EV biogenesis. Thus, we became interested in proteins upstream of *Vamp33*. Among the Rab-GTPases identified, we found only one sequence with identity to both mammalian *rab27a* and *rab27b* in the *I. scapularis* genome (Fig. 1, Additional file 1: Table S2). Given this observation, we chose to examine the tick *rab27* gene.

### Importance of Rab27 for EV biogenesis in *I. scapularis*

**Fig. 2:**
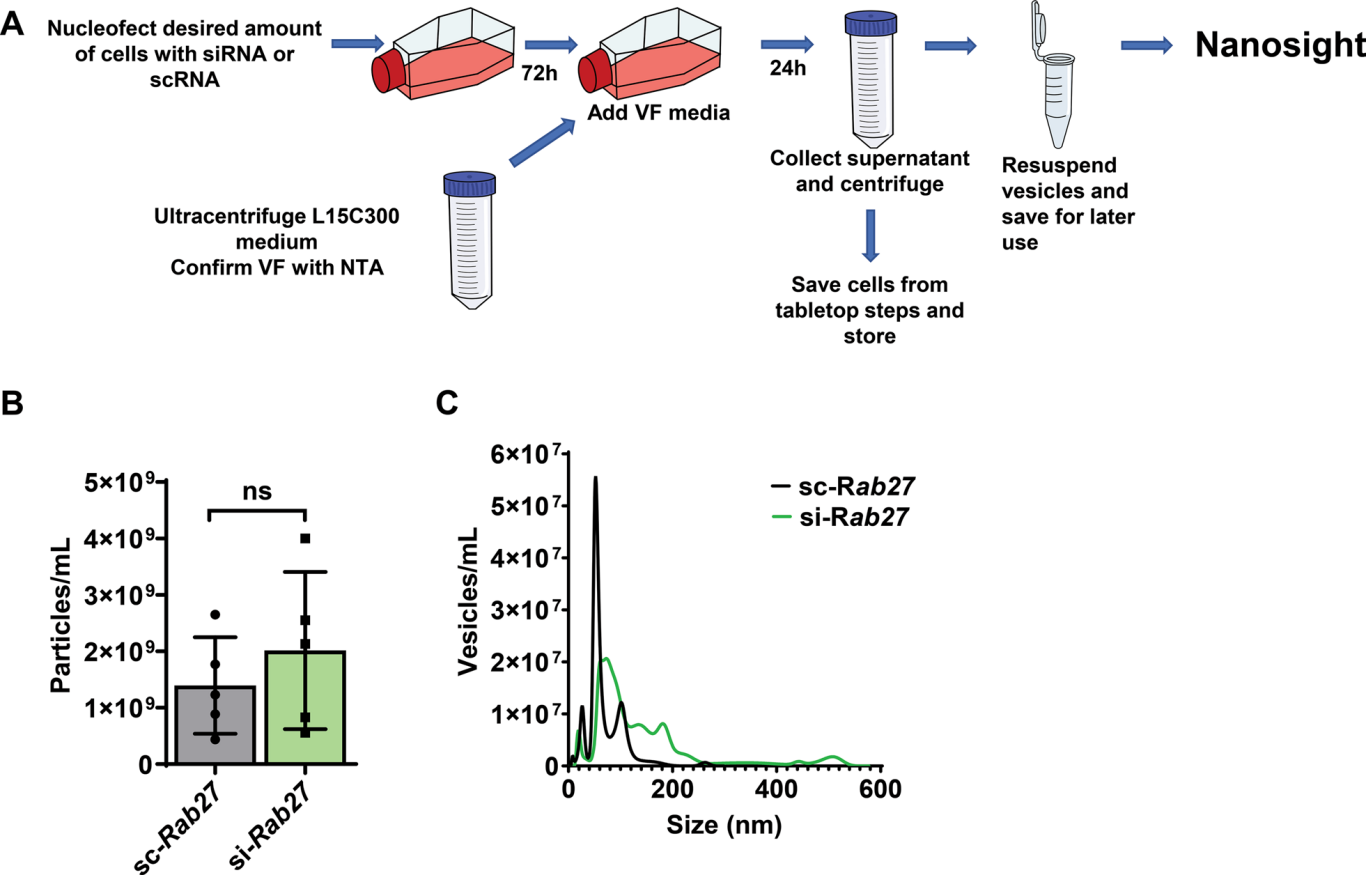
*rab27* silencing shifts the size of EVs being released by tick cells. **A** Experimental schematic for vesicle collection from tick cells. Tick ISE6 cells were nucleofected with small interfering (si-*rab27*) (green) or scrambled (sc-*rab27*) (gray) RNA for tick *rab27*. After 72 h, the cell culture media was replaced with vesicle free (VF) media. 24-h later, vesicles were collected through a series of tabletop and ultracentrifuge steps and measured using a nanosight machine. Graphs are representative of one two independent experiments. **B** Total vesicle amount released by tick cells does not change between the si-*rab27* or sc-*rab27* treatments. Statistical significance was evaluated by an unpaired, two-tailed *t*-test. Mean ± standard deviation (SD) is plotted. ns = not significant, *P* > 0.05. **C** Distribution of vesicles collected from sc-*rab27* treated cells and compared to the si-*rab27* treatment ranging from 0 to 600 nm

We opted to investigate the function of Rab27 during the formation of EVs in the tick. Thus, we nucleofected tick ISE6 cells with tick Rab27 siRNA or scrambled RNA (scRNA) (Fig. 2A). We then measured their size and concentration with Nano-particle tracking analysis (NTA) and observed that the total release of vesicles in tick cells did not change between silenced and scrambled treatments (Fig. 2B). However, the size range of the *rab27* silenced vesicles shifted to larger vesicles compared to the scrambled treatment (Fig. 2C). The sc-*rab27*-treated tick cells mostly produced vesicles between 0 and 300 nm. Conversely, si-*rab27*-treated tick cells produced vesicles up to ≤ 600 nm. Collectively, the shift in size indicated that Rab27 is involved in tick EV biogenesis.

### Silencing *Rab27* impacts *I. scapularis* feeding

**Fig. 3:**
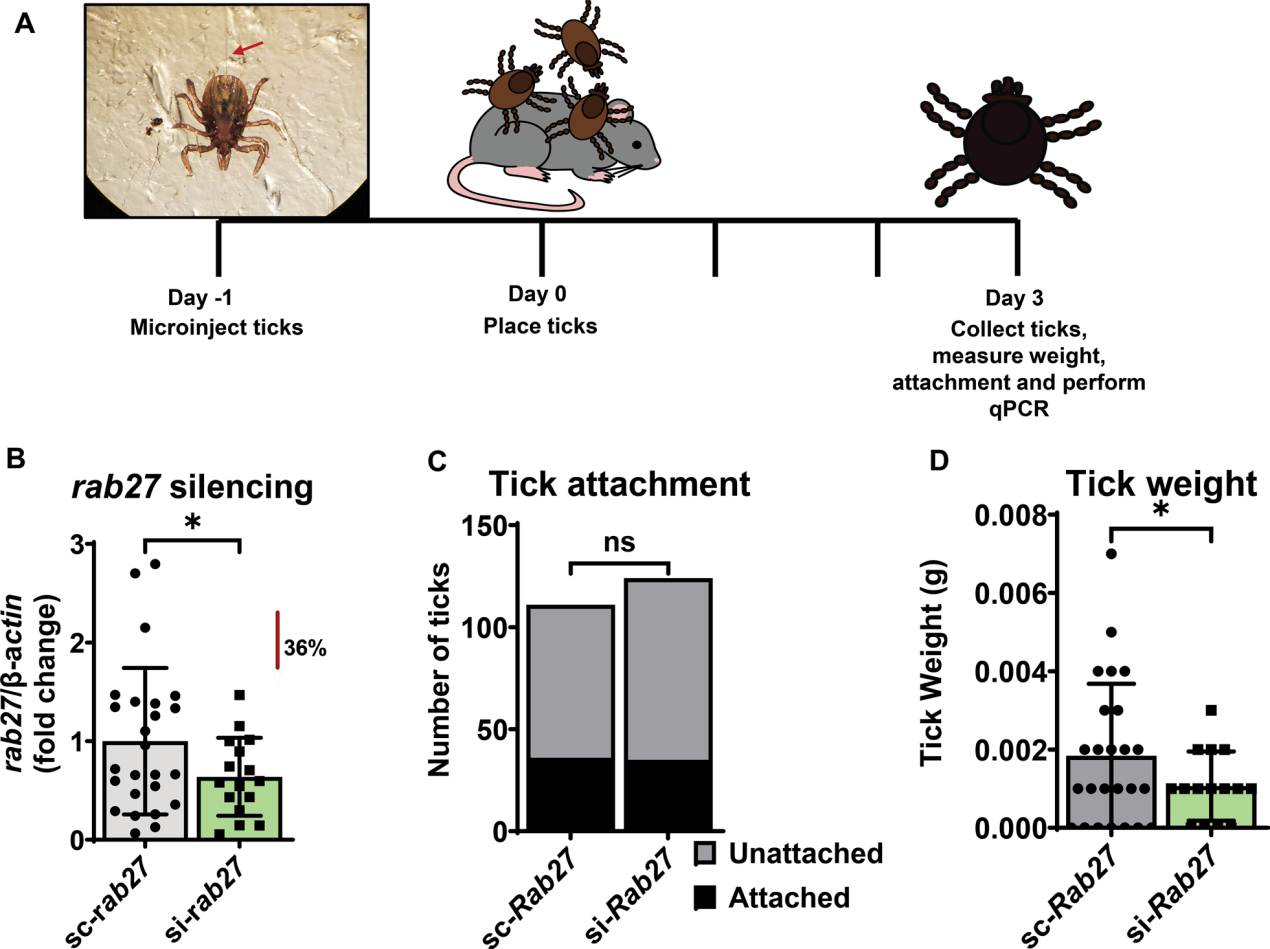
*rab27* silencing affects the fitness of *Ixodes scapularis* nymphs during hematophagy. **A** *Ixodes scapularis* nymphs were microinjected with small interfering (si-*rab27*)(green) or scrambled (sc-*rab27*)(gray) RNA, incubated for 24 h, and then placed on uninfected mice. **B** *rab27* expression relative to actin after silencing. The graph represents three independent experiments combined. si-*rab27* (*n* = 16). sc-*rab27* (*n* = 25). Mean ± standard deviation (SD) is plotted. Statistical significance was evaluated by an unpaired, two-tailed *t*-test with Welch’s correction. *P < 0.05. **C** *rab27* silencing effect on tick attachment. The graph represents three independent experiments combined. A Fisher’s exact test was performed to determine statistical differences. ns = not significant, *P* > 0.05. **D** Tick weight after *rab27* silencing. The graph represents three independent experiments combined. si-*rab27* (*n* = 16). sc-*rab27* (*n* = 25). Mean ± SD is plotted. Statistical significance was evaluated by an unpaired, two-tailed *t*-test with Welch’s correction. *P < 0.05. Ticks recorded as a weight of 0 mg were below the limit of detection of our scale (1 mg)

Next, we wanted to determine the effect of *rab27* silencing in vivo. We microinjected *I. scapularis* nymphs with either si-*rab27* or sc-*rab27* (Fig. 3A). After 24 h, ticks were placed on C57BL/6 mice for 3 days. Ticks that did not attach and fell from mice were recovered from the water trap and counted each day. On the 3rd day, we removed ticks that remained attached to the mouse, weighed them, and performed RNA extraction and then qPCR to confirm silencing. We observed significant silencing in the RNAi-treated ticks compared to the scrambled controls (Fig. 3B). Silencing *rab27* did not affect tick attachment (Fig. 3C); however, ticks in both the control and treatment groups struggled to attach. The health of the ticks upon receipt or experience of the microinjector may influence attachment rate. Additionally, ticks freely roamed on the mouse. The mouse may disturb ticks during grooming or possibly eat them, leading to decreased attachment. Importantly, *rab27* silenced ticks weighed less than their scrambled counterparts (Fig. 3D), indicating that Rab27 is important for *I. scapularis* hematophagy.

### *rab27* silencing reduces acquisition of *A. phagocytophilum* by *I. scapularis*

**Fig. 4:**
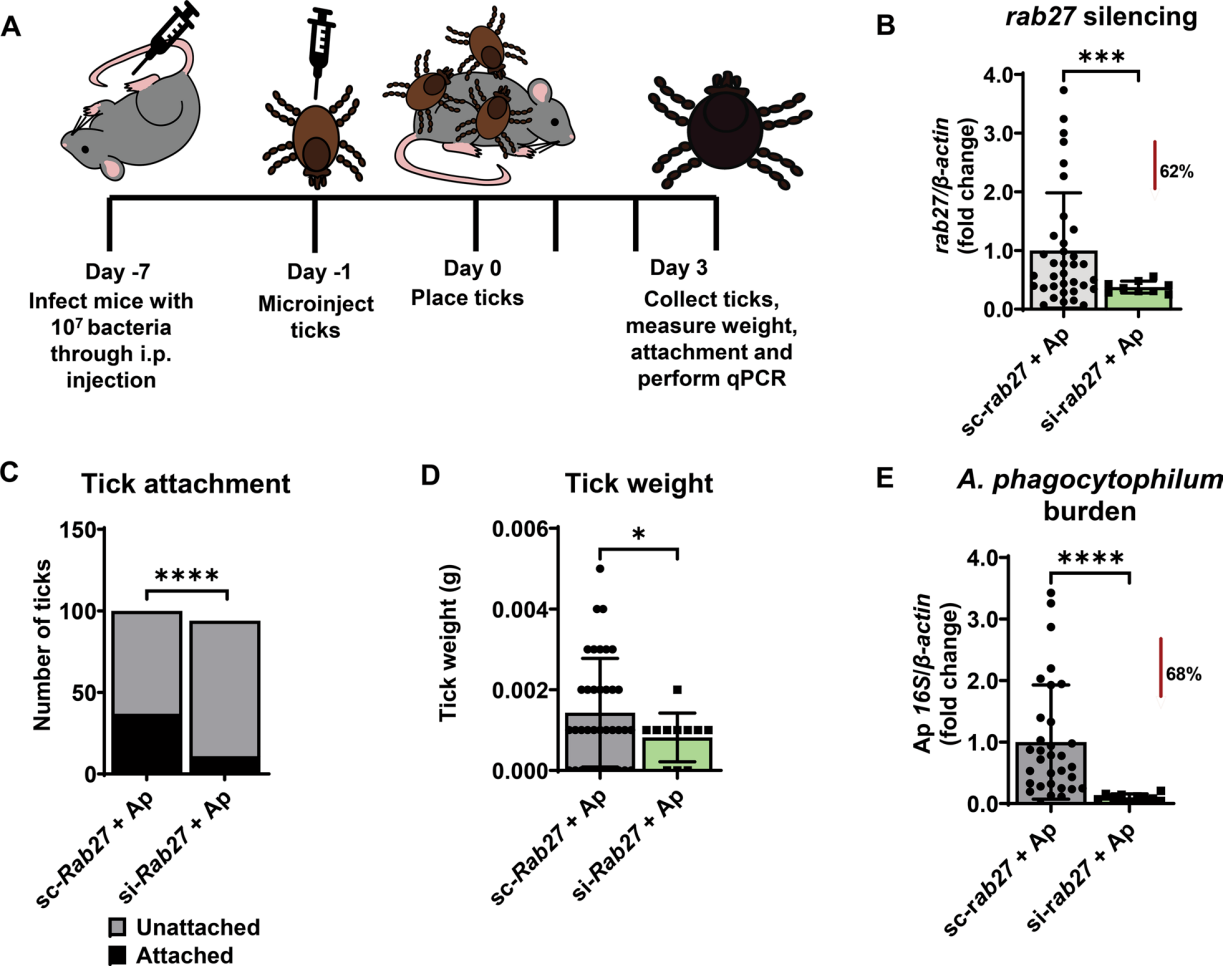
*rab27* silencing impacts *Anaplasma phagocytophilum* acquisition during blood feeding of *Ixodes scapularis* nymphs. **A** C57BL/6 mice were intraperitoneally (i.p.) injected with 10^7^ *A. phagocytophilum* (Ap) 7 days prior to tick placement. Ticks were microinjected with small interfering (si-*rab27*) (green) or scrambled (sc-*rab27*)(gray) RNA, incubated for 24 h, and then placed on infected mice. Ticks were recovered daily from a water trap. Remaining ticks attached to the mouse at day 3 were removed by forceps. **B** *rab27* silencing in *I. scapularis* ticks placed on Ap infected mice. The graph represents three independent experiments combined. si-*rab27* (*n* = 9). sc-*rab27* (*n* = 35). Mean ± standard deviation (SD) is plotted. Statistical significance was evaluated by an unpaired, two-tailed *t*-test with Welch’s correction. ***P < 0.0005. **C** Impact of *rab27* silencing on tick attachment. The graph represents three independent experiments combined. Fisher’s exact test was performed to determine statistical differences. ****P < 0.0001. **D** Effect of *rab27* silencing on tick weight. The graph represents three independent experiments combined. si-*rab27* (*n* = 11). sc-*rab27* (*n* = 37). Statistical significance was evaluated by an unpaired, two-tailed *t*-test with Welch’s correction. *P < 0.05. Ticks recorded as a weight of 0 mg were below the limit of detection of our scale (1 mg). **E** Impact of *rab27* silencing on Ap acquisition. The graph represents three independent experiments combined. si-*rab27* (*n* = 10). sc-*rab27* (*n* = 32). Mean ± SD is plotted. Statistical significance was evaluated by an unpaired, two-tailed *t*-test with Welch’s correction. ****P < 0.0001

Intracellular bacteria co-opts Rab GTPases to create a niche for their survival. Prior research has also shown that ISE6 cells infected with *A. phagocytophilum* produce an increased number of vesicles compared to uninfected cells [13]. Given that Rab27 is important for tick vesicle production and feeding and is involved in the life cycle of some intracellular bacteria, we explored how silencing of *rab27* affected pathogen acquisition. C57BL/6 mice were intraperitoneally injected with 10^7^ *A. phagocytophilum* 7 days before tick infestation (Fig. 4A). One day prior to tick placement, *I. scapularis* nymphs were microinjected with either si-*rab27* or sc-*rab27*. After 24-h incubation, we placed the microinjected ticks on the infected mice and allowed them to feed for 3 days. Unattached ticks were recovered from the water trap daily. After 3 days, the attached ticks were removed, weighed, and used for RNA extraction and qPCR determination of silencing. qPCR analysis detected significant levels of silencing compared to the scrambled controls (Fig. 4B). However, unlike the ticks fed on uninfected mice, significantly fewer *rab27* silenced ticks remained attached to the infected mice compared to the *rab27* scrambled treatment (Fig. 4C). As in our experiments with ticks on naïve mice, tick attachment may be impacted by mouse grooming, tick health upon arrival, or the microinjector experience. Like our previous results, *rab27* silencing led to decreased blood meal intake (Fig. 4D), which resulted in a significant reduction in *A. phagocytophilum* relative levels compared with the scrambled ticks (Fig. 4E). These results show that when *I. scapularis* feeds on an infected host, Rab27 is important for tick fitness, including attachment. It remains unclear whether the decrease observed in *A. phagocytophilum* burden was due to the diminished hematophagy in *Rab27*-silenced ticks or a direct effect on pathogen establishment.

## Discussion

Ticks are responsible for most vector-borne diseases in the US. Due to their multiple life stages, feeding requirements, and limited genetic tools, uncovering their cellular biology has been challenging. After silencing *rab27* in ISE6 cells, we observed a shift in vesicle size showing that a protein known to be involved in EV biogenesis in mammals is important for *I. scapularis* EV biogenesis and fitness. Mammals have two isoforms of Rab27, whereas *I. scapularis* ticks only have one present in their genome. In mammals, Rab27a and Rab27b have roles in separate stages of EV biogenesis [18]. Rab27a orchestrates the movement of the MVE to the plasma membrane of the cell. Rab27b is important for the docking of the MVE to the plasma membrane prior to exosome release [18]. Despite the change in vesicle size, it remains unclear whether the tick Rab27 protein functions in a similar manner to its roles identified in mammals. It is also conceivable that the tick Rab27 protein may function distinctly from what has been described in mammalian literature. Further research will need to be completed to determine tick Rab27’s specific role in EV biogenesis.

We observed that Rab27 is important for proper tick feeding and acquisition of *A. phagocytophilum*. Because silencing *rab27* results in an alteration of vesicle size, it is plausible that the vesicles do not perform their function during tick feeding correctly, resulting in reduced fitness. This is possibly due to altered surface proteins, improper trafficking, or disrupted formation. Alternatively, the cargo in the vesicle after *rab27* silencing may have changed, impacting vesicle function and tick fitness. Incorrect cargo may not influence the tick-host interface as it should or not interact at all, resulting in impaired feeding. We have not distinguished whether the reduced acquisition of *A. phagocytophilum* was due to the impediment of tick feeding after *rab27* silencing or, alternatively, if Rab27 is needed for the life cycle of *A. phagocytophilum* in ticks. Previous work has shown that the tick-borne pathogens *A. phagocytophilum*, *Anaplasma marginale*, and *Ehrlichia chaffeensis* are associated with several Rab GTPases [23–27]. Additionally, *E. chaffeensis* utilize Rabs during infection by recruiting them to the occupied vacuole and delaying endosome maturation [27, 28]. Furthermore, recent findings show that Rab27 is important for the egress of *A. phagocytophilum* from mammalian cells [29]. Whether this manipulation occurs in ticks remains elusive.

We observed that tick attachment was not affected when silencing *rab27* and feeding on uninfected mice. However, when *rab27* silenced ticks were fed on *A. phagocytophilum*-infected mice, silenced ticks attached significantly less than scrambled treated ticks. Prior research from our laboratory showed that when tick cells were infected with *A. phagocytophilum*, they secreted increased EVs compared to uninfected or *Borrelia burgdorferi*-treated cells [13]. Since silencing *rab27* disrupts EV biogenesis and *A. phagocytophilum* causes an increase of EV production, the effects of *rab27* silencing may become more pronounced when feeding ticks on infected mice, thus resulting in an attachment difference. Although given that tick attachment can be influenced by multiple factors, we are reticent to draw a firm conclusion from the attachment data without further experimentation. Moreover, *I. scapularis* salivary vesicle content has been examined in naïve ticks but not under infection conditions [13]. Given *A. phagocytophilum* infection increases EV secretion in tick cells, we anticipate that infection alters vesicle number, size, cargo, and post-translation protein modifications, but further study is required [13]. The molecular interactions between Rab27 and *A. phagocytophilum* are not defined in the tick *I. scapularis*. Future research could uncover an important relationship between tick EV biogenesis proteins and tick-borne diseases.

With recent advancements in the technology available to the vector-borne disease community, such as Clustered Regularly Interspaced Palindromic Repeats (CRISPR) in ticks [30], and ectopic expression in tick cells [31], future work can aim to unravel Rab27’s exact mechanism of action as well as its interaction with important tick-borne pathogens, such as *A. phagocytophilum* [32]. Furthermore, it is unclear whether Rab27 is important for tick fitness in other species. Research regarding vesicle formation in ticks and their importance for acquisition or transmission of tick-borne disease could reveal future targets to mitigate the spread of illnesses unraveling a previous unexplored aspect of vector biology. Overall, we have shown that Rab27 is important for tick EV biogenesis and fitness in *I. scapularis*. We shed light on a previously unstudied aspect of tick biology.

## Conclusions

Disrupting *rab27* impairs tick feeding and *A. phagocytophilum* acquisition during a blood meal. Additionally, silencing *rab27* in tick cells results in a shift of extracellular vesicle size. Rab27 appears to play a role in tick EV biogenesis. Future research and technological advancements will allow for further study into tick biology, the specific role of Rab27, and its importance for tick-borne disease transmission.

### Supplementary Information

**Additional file 1: Table S1.** The primers used in this study. **Table S2.** Alignment of protein sequences related to EV biogenesis. **Table S3.** Reagents used in this study.

## Data Availability

All datasets have been included with this article.

## Abbreviations

EV: Extracellular vesicle
ESCRT: Endosomal sorting complex required for transport
SNARE: Soluble N-ethylmaleimide-sensitive factor attachment receptor
ILVs: Intraluminal vesicles
MVE: Multivesicular endosome
BLASTp: Basic Local Alignment Search Tool for proteins
NTA: Nanoparticle tracking analysis
siRNA: Small interfering RNA
scRNA: Scrambled RNA
i.p.: Intraperitoneally
d.p.i.: Days post infection
